# Impact of Meat Consumption, Preparation, and Mutagens on Aggressive Prostate Cancer

**DOI:** 10.1371/journal.pone.0027711

**Published:** 2011-11-23

**Authors:** Sanoj Punnen, Jill Hardin, Iona Cheng, Eric A. Klein, John S. Witte

**Affiliations:** 1 Department of Urology, University of California San Francisco, San Francisco, California, United States of America; 2 Department of Epidemiology and Biostatistics, University of California San Francisco, San Francisco, California, United States of America; 3 Institute for Human Genetics, Helen Diller Family Comprehensive Cancer Center, University of California San Francisco, San Francisco, California, United States of America; 4 Epidemiology Program, University of Hawai'i Cancer Center, University of Hawai'i, Honolulu, Hawai'i, United States of America; 5 Cleveland Clinic Glickman Urological and Kidney Institute and Taussig Cancer Institute, Cleveland, Ohio, United States of America; Florida International University, United States of America

## Abstract

**Background:**

The association between meat consumption and prostate cancer remains unclear, perhaps reflecting heterogeneity in the types of tumors studied and the method of meat preparation—which can impact the production of carcinogens.

**Methods:**

We address both issues in this case-control study focused on aggressive prostate cancer (470 cases and 512 controls), where men reported not only their meat intake but also their meat preparation and doneness level on a semi-quantitative food-frequency questionnaire. Associations between overall and grilled meat consumption, doneness level, ensuing carcinogens and aggressive prostate cancer were assessed using multivariate logistic regression.

**Results:**

Higher consumption of any ground beef or processed meats were positively associated with aggressive prostate cancer, with ground beef showing the strongest association (OR = 2.30, 95% CI:1.39–3.81; P-trend = 0.002). This association primarily reflected intake of grilled or barbequed meat, with more well-done meat conferring a higher risk of aggressive prostate cancer. Comparing high and low consumptions of well/very well cooked ground beef to no consumption gave OR's of 2.04 (95% CI:1.41–2.96) and 1.51 (95% CI:1.06–2.14), respectively. In contrast, consumption of rare/medium cooked ground beef was not associated with aggressive prostate cancer. Looking at meat mutagens produced by cooking at high temperatures, we detected an increased risk with 2-amino-3,8-Dimethylimidazo-[4,5-f]Quinolaxine (MelQx) and 2-amino-3,4,8-trimethylimidazo(4,5-f)qunioxaline (DiMelQx), when comparing the highest to lowest quartiles of intake: OR = 1.69 (95% CI:1.08–2.64;P-trend = 0.02) and OR = 1.53 (95% CI:1.00–2.35; P-trend = 0.005), respectively.

**Discussion:**

Higher intake of well-done grilled or barbequed red meat and ensuing carcinogens could increase the risk of aggressive prostate cancer.

## Introduction

Prostate cancer is the most common non-skin cancer and second most common cause of cancer related death in men in the United States [1]. The estimated lifetime risk of prostate cancer in white and African American males is 17.6% and 20.6%, respectively [1]. Known risk factors for prostate cancer include age, family history, ethnicity, and a number of genetic variants. While prostate cancer is highly heritable, geographic variation in the incidence of prostate cancer and the increased risk conferred to men who relocate from low to high risk countries suggest that environmental factors may also play a role in this common but complex disease [2].

Numerous epidemiological studies have assessed the impact of dietary factors on prostate cancer, and those investigating meat consumption have given mixed results [3]–[13]. Several studies have reported positive associations between red meat consumption and prostate cancer risk [7], [8], [12], [14]–[16]. For example, a large cohort study (N = 175,343 men) found that increased consumption of red meat was associated with overall prostate cancer and advanced prostate cancer [17]. However, a recent meta-analysis failed to detect a positive association between total red meat consumption and prostate cancer (RR = 1.00, 95% CI: 0.96–1.05) and found only a weak association between processed meat consumption and prostate cancer (RR = 1.05, 95% CI: 0.99–1.12) [18]. Moreover, in a 2007 report on diet and cancer, the World Cancer Research Fund/American Institute of Cancer Research concluded that evidence for an association between various meats and prostate cancer risk was “limited-no conclusion” [19].

One possible explanation for these equivocal results is that any potential meat association might be restricted to more advanced or aggressive disease. Prostate cancer is extremely heterogeneous: some tumors remain latent while others are more aggressive and rapidly progress. Studies focused on the more aggressive subtypes of prostate tumors have detected associations with meat intake [17], [20]–[22]. Nevertheless, this possibility remains muddled as some studies looking at advanced/aggressive disease have not seen an association between meats and prostate cancer [5], [23]–[25].

Another possible explanation for these equivocal results is that the key exposure is not just meat intake, but also how it is prepared. Recent studies looking at the doneness level or charred index of meat preparation have suggested an increased risk of prostate cancer from meats cooked at high temperatures, such as pan-frying or grilling [22], [26], [27]. This is believed to occur from the production of carcinogenic heterocyclic amines (HCA) and polycyclic aromatic hydrocarbons such as 2-amino-3,8-dimethylimidazo(4,5-f)quinoxaline (MelQx), 2-amino-3,4,8-trimethylimidazo(4,5-f)quinoxaline (DiMelQx), 2-amino-1-methyl-6-pheylimidazo(4,5-b)pyridine (PhIP) and benzo(a)pyrene (BaP), which occur when meat is cooked at high temperatures.

Here we further investigate the possibility that meat associations depend on prostate cancer aggressiveness and cooking methods. In particular, we studied the consumption of overall versus grilled meat, how well-done the latter was prepared, and the ensuing production of heterocyclic amines in a case-control study of men with aggressive prostate cancer. Given the public heath impact of prostate cancer, any dietary and chemo-preventive strategies to reduce the economic, emotional, and physical burden of prostate cancer would be critically important.

## Materials and Methods

### Study Subjects

Between 2001 and 2004, aggressive incident prostate cancer cases and frequency- matched controls were recruited from the major medical institutions in Cleveland, Ohio (The Cleveland Clinic, University Hospitals of Cleveland, and their affiliates). Physicians at these institutions see a large majority of men diagnosed with prostate cancer in the Greater Cleveland area. Hence, while the sample was not formally population-based, the cases were fairly representative of men diagnosed with prostate cancer in the Cleveland region.

The cases were newly diagnosed with histologically confirmed disease, with any one of the following: Gleason score ≥7; tumor stage ≥T2c; or a prostate-specific antigen level greater than 10 ng/ml at diagnosis. Cases were contacted as quickly as possible following diagnosis with prostate cancer (median time between diagnosis and recruitment, 4.7 months). Studying more aggressive cases allowed us to focus on men with the most clinically relevant disease. Case diagnoses were verified from medical record review and Gleason scores were based on pathology reports from radical prostatectomy specimens when available and otherwise from biopsy specimens. A total of 501 cases were fully recruited into the study (e.g., provided biospecimens for other research); 470 completed the Food Frequency Questionnaire (FFQ) and 466 completed the meat preparation questionnaire and are included here.

To ensure the controls were representative of the source population of cases, controls were men who underwent annual medical exams at the collaborating medical institutions. Controls had no diagnosis of prostate cancer or any other non-skin cancer. At the study entry all controls underwent prostate cancer screening with serum PSA testing and follow-up if their PSA was inflated. If a value of 4.0 ng/ml or greater was attained then a formal evaluation for prostate cancer by a urologist was undertaken. Depending on the evaluation, a biopsy of the prostate for histological diagnosis was preformed. Follow up of 50 control patients with a PSA greater than 4.0 ng/ml led to the diagnosis of two new prostate cancer cases. Both cases met the criteria for aggressive prostate cancer and were subsequently included as cases in this study. Controls were frequency matched to cases with respect to age (within five years), ethnicity, and medical institution. Data was collected on demographic, clinical, and histological measures during an in-person computer aided interview. A total of 538 controls were recruited into the study. 512 of these completed the FFQ and 511 completed the meat preparation questionnaire and are included here.

Ethics approval for this study was obtained from the Institutional Review Board at the University of California, San Francisco committee on human research as well at all institutions/hospitals where participants were recruited and human experimentation was conducted (University Hospitals of Cleveland, and their affiliates - Cleveland Clinic Foundation, Case Western Reserve University, and the Henry Ford Health Systems). All patients in this study provided written informed consent.

### Dietary Assessment of Meat Consumption

Information regarding diet was collected using a validated semi-quantitative food frequency questionnaire (FFQ) developed by the Nutrition Assessment Shared Resource of the Fred Hutchinson Cancer Research Center with a particular focus on prostate cancer (Figure S1) [28], [29]. The FFQs were completed by the cases and controls at enrollment into the study. The cases were asked to recall their food consumption over the year prior to their diagnosis of prostate cancer, while the controls were asked to recall their food consumption during the previous year. The FFQ ascertained information on various types of foods, including a range of meats and the frequency of consumption. A supplemental questionnaire asking about intake of grilled or barbequed meats and red meat doneness levels was added to the FFQ and completed by the study subjects at the same time; this information allowed estimation of HCA intake [30]. Questionnaires were mailed to study subjects and self-administered. These questionnaires were then scanned at the Fred Hutchinson Cancer Research Center.

HCA and polycyclic aromatic hydrocarbon consumption levels were estimated for red meats by multiplying the grams of intake prepared in a particular manner by the appropriate mutagen content provided by the National Cancer Institute's CHARRED database (http://charred.cancer.gov). The following mutagen levels were estimated: MelQx (2-amino-3,8-dimethylimidazo(4,5-f)quinoxaline); DiMelQx (2-amino-3,4,8-trimethylimidazo(4,5-f)quinoxaline); PhIP (2-amino-1-methyl-6-pheylimidazol(4,5-b)pyridine); and BaP (benzo(a)pyrene).

For these analyses, we excluded 21 subjects because of implausible values for total calorie intake (<500 or >5,000 kcal/d), leaving 470 cases and 512 controls for the total meats analysis. In addition 5 subjects did not complete the grilled meat and food doneness table, and so are excluded from those analyses.

### Statistical Analysis

We examined the association between total and grilled meat intake, red meat doneness, and HCAs and aggressive prostate cancer using logistic regression models. We evaluated the main effects of individual meats and red meats combined. Meats were combined based on the way they were grouped on the food frequency questionnaire. Meat intake was categorized into three or four categories of increasing consumption based on the distribution of servings per week among control patients. HCAs were categorized into approximate quartiles based on their distribution among controls. Odds ratios and 95% confidence intervals comparing increasing weekly servings of meat to no meat consumption were reported. We also examined the joint effect of red meat consumption and doneness level. P-trend values were calculated with the exposures modeled continuously across all categories (i.e., assigning values of 1, 2, 3, and 4 for individuals within each of the four quartiles, respectively).

All logistic regression models were adjusted for the matching variables (age, ethnicity, and medical institution) as well as for total energy intake, incorporating calories as a continuous variable. Furthermore, to evaluate potential confounding due to other factors that might impact consuming more meats and prostate cancer screening, we examined the impact of the following covariates: family history of prostate cancer in first degree relatives (prostate cancer in brother and/or father), smoking (never, former, or current), body mass index (kilograms per meter squared (kg/m^2^)), prior history of PSA testing for prostate cancer (never/once/twice or more), education level (4 categories of levels of schooling), and omega 3 fatty acid intake. None of these covariates materially influenced the associations between meat, doneness, or HCA and prostate cancer (always resulting in a <10% change in the corresponding regression coefficients) and are thus excluded from our final models. All analyses were undertaken with SAS software (version 9.1; SAS Institute).

## Results

The demographic and clinical characteristics of the study subjects are presented in Table 1. Cases reported lower education, a higher frequency of family history of prostate cancer, and a previous history of PSA testing than controls. The average PSA at diagnosis for cases was 14.1 ng/mL and 85.3% of the cases had a Gleason score ≥7. Mean dietary intake of total calories, total and grilled red meats, and grilled chicken were statistically significantly higher in cases than controls (Table 2). With respect to meat mutagens, cases had a higher mean intake of MelQx and DiMelQx, but not PhIP or BaP (Table 2).

**Table 1:** Characteristics of case-control study population of aggressive prostate cancer.

Characteristic^a^	Cases (n = 470)		Controls (n = 512)		P-value^b^
Age (years), mean (SD^c^)	65.8	(8.3)	65.9	(8.5)	0.86
Ethnicity, n (%)					0.63
African-American	78	(16.6)	91	(17.8)	
Caucasian	392	(83.4)	421	(82.2)	
Education, n (%)					<0.001
<12 years	43	(9.1)	45	(8.8)	
12 years or high school	105	(22.3)	68	(13.3)	
Some college	98	(20.9)	91	(17.8)	
≥College graduate	223	(47.4)	306	(59.8)	
Family history of prostate cancer^d^, n (%)					<0.0001
Negative	359	(76.4)	452	(88.3)	
Positive	110	(23.4)	55	(10.7)	
Smoking, n (%)					0.35
Never	192	(40.9)	208	(40.6)	
Former	224	(47.7)	255	(49.8)	
Current	53	(11.3)	45	(8.8)	
Body mass index (kg/m^2^) mean (SD)	26.2	(3.7)	26.4	(3.7)	0.55
Prior history of PSA test, n (%)					0.02
Never	100	(21.3)	113	(22.1)	
Once	53	(11.3)	68	(13.3)	
Twice or more	296	(63.0)	286	(55.9)	
Serum PSA value (ng/ml), mean (SD)	14.1	(24.8)	1.7	(1.7)	<0.0001
Clinical stage, n (%)					
T1	294	(62.6)			
T2	119	(25.3)			
T3 & T4	39	(8.3)			
Histologic tumor grade: Gleason score n (%)					
≤6	69	(14.7)			
7	298	(63.4)			
≥8	103	(21.9)			

a Some totals do not add to 100 percent due to missing data.

b From T-test comparing means, or chi-square tests comparing counts.

c SD, standard deviation.

d Positive family history of prostate cancer was defined as prostate cancer in a first degree relative.

**Table 2:** Intake of calories, total meats, meats prepared by grilling or barbecue, and meat mutagens in aggressive prostate cancer case-control study population.

Energy, Meat, and Mutagens	Cases		Controls		P-value^b^
	Mean	SD^a^	Mean	SD	
**Calories**	2,278	(879)	2,080	(787)	<0.001
**Total Meats** c					
Beef, pork, ham, and lamb	2.2	(1.9)	1.9	(1.9)	0.02
Ground meat: hamburgers and meatloaf	1.3	(1.2)	1.0	(1.0)	<0.001
Chicken and turkey	1.2	(1.2)	1.3	(1.2)	0.06
Regular hotdogs and sausage	0.4	(0.4)	0.7	(0.6)	0.35
Bacon and breakfast sausage	1.1	(1.8)	0.9	(1.3)	0.03
Lunch meats: ham, turkey and lowfat bologna	1.4	(1.8)	1.3	(1.7)	0.57
Other lunch meat: bologna, salami and Spam	0.6	(0.5)	1.2	(1.1)	0.02
Low or reduced fat hot dogs and sausage	0.2	(0.6)	0.2	(0.5)	0.69
Liver, chicken liver and organ meats	0.2	(0.6)	0.1	(0.3)	0.008
**Grilled or Barbecued Meats** c ^,^ d					
Beef	1.0	(1.4)	0.8	(1.1)	0.007
Hamburger	1.4	(1.9)	1.0	(1.5)	<0.001
Pork	0.6	(0.8)	0.5	(0.8)	0.31
Hot Dogs	0.2	(0.4)	0.2	(0.5)	0.81
Chicken	1.4	(1.8)	1.2	(1.6)	0.02
**Meat Mutagens** e					
MelQx	46	(53)	38	(53)	0.05
DiMeIQx	1.8	(3.0)	1.3	(2.7)	0.02
PhIP	203	(273)	185	(291)	0.40
BaP	89	(110)	89.2	(103)	0.12

a SD, standard deviation.

b From T-test comparing means.

c Means given in servings per week or ng/week for mutagens.

d N = 469 cases and 508 controls due to missing values.

e MelQx: 2-amino-3,8-dimethylimidazo(4,5-f)quinoxaline;

DiMelQx: 2-amino-3,4,8-trimethylimidazo(4,5-f)quinoxaline;

PhIP: 2-amino-1-methyl-6-pheylimidazol(4,5-b)pyridine;

BaP: benzo(a)pyrene.

The associations between overall meat intake and aggressive prostate cancer are given in Table 3. Odds ratios and 95% confidence intervals are provided for increasing levels of meat consumption based on the distribution of servings of meat per week among control patients. Meats were grouped based on how they were asked about on the food frequency questionnaire. Higher intake of ground meat, liver, and processed meats were associated with an increased risk of aggressive prostate cancer (Table 3). For ground meat (i.e., hamburgers) the adjusted odds ratios (OR; 95% CI) comparing the second, third, and fourth categories to the first were 1.59 (1.00–2.52), 1.78 (1.09–2.89), and 2.30 (1.39–3.81), respectively (P-trend = 0.002). For liver and processed meats, the ORs comparing the highest to lowest categories were 2.24 (1.29–3.88; P-trend = 0.02) and 1.57 (1.11–2.21; P-trend = 0.001), respectively.

**Table 3:** Association between meat consumption and risk of aggressive prostate cancer.

Meat	Servings per Week^a^				*P*-trend^d^
	None	Low	Medium	High	
**Beef, pork, ham and lamb**					
Case/Control Median^b^	0/0	0.58/0.58	2.00/2.00	3.46/3.46	
Cases/Controls (N)	34/47	105/125	170/200	161/140	
Odds Ratios^c^ (95% CIs)	1.00	1.13 (0.67,1.89)	1.03 (0.63, 1.70)	1.25 (0.74, 2.11)	0.43
**Ground meat:hamburgers & meatloaf**					
Case/Control Median^b^	0/0	0.58/0.58	1.00/1.00	2.00/2.00	
Cases/Controls (N)	35/70	162/199	123/130	150/112	
Odds Ratios^c^ (95% CIs)	1.00	1.59 (1.00, 2.52)	1.78 (1.09, 2.89)	2.30 (1.39, 3.81)	0.002
**Chicken and turkey**					
Case/Control Median^b^	0.25/0.25	0.58/0.58	1.00/1.00	2.00/2.00	
Cases/Controls (N)	92/101	135/120	107/103	135/188	
Odds Ratios^c^ (95% CIs)	1.00	1.23 (0.84, 1.79)	1.08 (0.73, 1.61)	0.70 (0.49, 1.02)	0.22
**Regular hot dogs and sausage**					
Case/Control Median^b^	0/0	0.25/0.25	0.58/0.58	1.00/1.00	
Cases/Controls (N)	167/221	123/111	115/110	64/70	
Odds Ratios^c^ (95% CIs)	1.00	1.35 (0.97, 1.88)	1.24 (0.88, 1.74)	0.97 (0.63, 1.48)	0.70
**Bacon and breakfast sausage**					
Case/Control Median^b^	0/0	0.25/0.25	0.58/0.58	2.00/2.00	
Cases/Controls (N)	116/143	69/86	95/92	190/191	
Odds Ratios^c^ (95% CIs)	1.00	0.93 (0.62, 1.40)	1.18 (0.80, 1.74)	1.11 (0.80, 1.54)	0.41
**Lunch meats: ham, turkey, bologna**					
Case/Control Median^b^	0/0	0.58/0.58	2.00/2.00	3.46/3.46	
Cases/Controls (N)	115/131	144/159	125/129	85/93	
Odds Ratios^c^ (95% CIs)	1.00	1.01 (0.72, 1.42)	1.01 (0.71, 1.45)	0.89 (0.60, 1.33)	0.63
**Lunch meat: bologna, salami, spam**					
Case/Control Median^b^	0/0	0.25/0.25	2.00/2.00		
Cases/Controls (N)	241/332	126/97	102/82		
Odds Ratios^c^ (95% CIs)	1.00	1.74 (1.27, 2.39)	1.57 (1.11, 2.21)		<0.001
**Low fat hot dogs and sausage**					
Case/Control Median^b^	0/0	0.25/0.25	0.58/0.58		
Cases/Controls (N)	347/376	48/54	74/82		
Odds Ratios^c^ (95% CIs)	1.00	0.97 (0.64, 1.48)	0.92 (0.64, 1.31)		0.63
**Liver, chicken liver and organ meats**					
Case/Control Median^b^	0/0	0.25/0.25	1.00/0.58		
Cases/Controls (N)	386/437	41/52	43/23		
Odds Ratios^c^ (95% CIs)	1.00	0.90 (0.58, 1.39)	2.24 (1.29, 3.88)		0.02

a Groupings defined so sufficient numbers are in each group given categorical nature of questionnaire.

b Median of category, servings per week for cases and controls.

c Odds ratios adjusted for age, race, institution, and energy intake.

d P-trend values from continuous model across categories.

Table 4 presents results restricted to meat intake that was grilled or barbecued at home or in a restaurant. Again, odds ratios and 95% confidence intervals are provided for increasing levels of meat consumption based on the distribution of servings per week among control patients. There were positive associations between increasing intake of barbecued beef, hamburger, chicken and aggressive prostate cancer. For beef and hamburger, the adjusted ORs comparing the highest categories to the lowest (i.e., no intake) were 1.61 (1.13–2.28; P-trend = 0.004) and 1.86 (1.28–2.71; P-trend = 0.001), respectively. Interestingly, when only considering increasing consumption of ground meat that was not grilled or barbecued, there was almost no association with aggressive prostate cancer (OR 1.23, 95% CI: 0.84–1.79, comparing the highest quartile of intake to the lowest). This suggests that the grilled or barbecued intake of beef and hamburger appeared to account for essentially all of the overall ground meat finding presented in Table 3, whereby the method of meat preparation may be a key factor here.

**Table 4:** Relationship between intake of grilled and barbecued meat and risk of aggressive prostate cancer.

Grilled Meat	Servings per Week^a^				*P*-Trend^d^
	None	Low	Medium	High	
**Beef**					
Case/Control Median^b^	0/0	0.25/0.25	0.88/0.88	2.00/1.63	
Cases/Controls (N)	131/200	85/87	124/108	129/113	
Odds Ratios (95% CI)^c^	1.00	1.50 (1.03, 2.19)	1.69 (1.19, 2.38)	1.61 (1.13, 2.28)	0.004
**Hamburger**					
Case/Control Median^b^	0/0	0.63/0.50	1.25/1.25	3.00/2.63	
Cases/Controls (N)	117/180	106/117	126/121	120/90	
Odds Ratios (95% CI)^c^	1.00	1.41 (0.99, 2.01)	1.58 (1.11, 2.24)	1.86 (1.28, 2.71)	0.001
**Pork**					
Case/Control Median^b^	0/0	0.25/0.25	0.76/0.88	1.63/1.63	
Cases/Controls (N)	195/255	95/88	96/84	83/81	
Odds Ratios (95% CI)^c^	1.00	1.39 (0.98, 1.97)	1.44 (1.01, 2.04)	1.21 (0.83, 1.75)	0.07
**Chicken**					
Case/Control Median^b^	0/0	0.63/0.63	1.26/1.26	3.00/3.00	
Cases/Controls (N)	128/170	107/130	103/96	131/112	
Odds Ratios (95% CI)^c^	1.00	1.14 (0.80, 1.61)	1.43 (0.99, 2.06)	1.48 (1.04, 2.11)	0.006
**Hot dogs**					
Case/Control Median^b^	0/0	0.25/0.25	0.63/0.63	1.00/1.00	
Cases/Controls (N)	289/336	67/75	83/54	30/43	
Odds Ratios (95% CI)^c^	1.00	1.04 (0.72, 1.50)	1.67 (1.13, 2.45)	0.71 (0.43, 1.18)	0.55

a Groupings defined so sufficient numbers are in each group given categorical nature of questionnaire.

b Median of category, servings per week for cases and controls.

c Odds ratios adjusted for age, race, institution, and energy intake.

d P-trend values from continuous model across categories.

Focusing on the grilled or barbecued beef and hamburger, we then investigated the effect of both consumption levels and doneness on aggressive prostate cancer (Table 5). Specifically, we cross-classified men based on whether they ate red meat that was cooked well/very well done versus rare/medium by their intake levels, and contrasted this with no intake. High consumption of well or very well cooked beef or hamburger was associated with an increased risk of aggressive prostate cancer compared to no consumption: OR = 2.16 (1.37–3.38) and OR = 2.04 (1.41–2.95), respectively. A slightly weaker but still noteworthy association was observed for low consumption of well or very well cooked beef and hamburger and aggressive prostate cancer: OR = 1.92 (1.29–3.38) and OR = 1.51 (1.06–2.14), respectively. In contrast, high or low consumption of rare or medium cooked red meat did not appear to be associated with aggressive prostate cancer, suggesting that doneness is more important than the absolute intake (Table 5).

**Table 5:** Association between consumption and doneness of grilled and barbecued beef and hamburger and aggressive prostate cancer.

		Rare/Medium Done^a^		Well & Very-Well Done		*P*-Trend^d^
Grilled Meat	None	Low Intake	High Intake	Low Intake	High Intake	
**Beef**						
Case/Control Median^b^	0/0	0.50/0.63	1.63/1.63	0.63/0.50	1.26/1.26	
Cases/Controls (N)	131/200	93/99	92/99	81/65	69/43	
Odds Ratios (95% CI)^c^	1.00	1.35 (0.94, 1.95)	1.29 (0.89, 1.88)	1.92 (1.29, 2.86)	2.16 (1.37, 3.38)	<0.001
**Hamburger**						
Case/Control Median^b^	0/0	0.63/0.63	2.00/2.00	0.63/0.63	2.25/2.00	
Cases/Controls (N)	117/180	60/65	47/59	113/114	128/87	
Odds Ratios (95% CI)^c^	1.00	1.35 (0.88, 2.07)	1.12 (0.71, 1.79)	1.51 (1.06, 2.14)	2.04 (1.41, 2.95)	<0.001

a Groupings defined so sufficient numbers are in each group given categorical nature of questionnaire.

b Median of category, servings per week for cases and controls.

c Odds ratios adjusted for age, race, institution, and energy intake.

d P-trend values from continuous model across categories.

With respect to meat mutagens produced by cooking at high temperatures, MelQX and DiMelQx were positively associated with increased risk of aggressive prostate cancer (Table 6). Comparing the highest quartile of MelQx consumption to the lowest quartile gave an OR = 1.69 (1.08–2.64; P-trend = 0.02). For DiMelQX, comparing the third and fourth quartiles of consumption to the first quartile gave ORs equal to 1.84 (1.22–2.77) and 1.53 (1.00–2.35) (P-trend = 0.005), respectively. In contrast, PhIP and BaP did not appear statistically significantly associated with aggressive prostate cancer risk (Table 6). Another model of the relation between mutagens and aggressive prostate cancer was created controlling for tomato products and cruciferous vegetables. The results of this model were not materially changed (data not shown).

**Table 6:** Association between meat mutagens and aggressive prostate cancer among men who consumed barbecued or grilled meat.

Mutagen^a^	Quartiles				*P*-trend^b^
	1	2	3	4	
**MelQx**					
Case/Control Median^c^	4.0/4.0	15.8/15.5	34.7/33.6	89.6/88.5	
Cases/Controls (N)	75/93	89/96	90/84	106/71	
Odds Ratios (95% CI)^d^	1.00	1.16 (0.76, 1.78)	1.31 (0.85, 2.03)	1.69 (1.08, 2.64)	0.02
**DiMeIQx**					
Case/Control Median^c^	0/0	0.7/0.5	4.6/1.6	5.5/4.4	
Cases/Controls (N)	163/195	44/45	82/54	71/50	
Odds Ratios (95% CI)^e^	1.00	1.21(0.76, 1.95)	1.84 (1.22, 2.77)	1.53 (1.00, 2.35)	0.005
**PhIP**					
Case/Control Median^c^	21.2/18.8	73.6/66.3	154.5/151.0	404.5/432.1	
Cases/Controls (N)	86/91	80/95	93/85	101/73	
Odds Ratios (95% CI)^e^	1.00	0.88 (0.57, 1.34)	1.10 (0.72, 1.69)	1.32 (0.86, 2.05)	0.13
Benzo[*a*]pyrene					
Case/Control Median^c^	3.3/3.3	24.1/20.0	82.4/76.0	186.6/173.5	
Cases/Controls (N)	81/97	89/86	93/83	97/78	
Odds Ratios (95% CI)^e^	1.00	1.22 (0.80, 1.87)	1.32 (0.87, 2.02)	1.34 (0.87, 2.07)	0.17

a Mutagens are:

MelQx: 2-amino-3,8-dimethylimidazo(4,5-f)quinoxaline;

DiMelQx: 2-amino-3,4,8-trimethylimidazo(4,5-f)quinoxaline (analyzed using 4 categories);

PhIP: 2-amino-1-methyl-6-pheylimidazol(4,5-b)pyridine;

b P-trend values across quartiles.

c Medians given in ng per week for cases and controls.

d Odds Ratios adjusted for age, sex, race, institution, and energy intake.

## Discussion

The key finding here was that higher consumption of red meat was positively associated with risk of aggressive prostate cancer. This result appeared primarily driven by red meat that was grilled or barbequed—especially when cooked well-done. Furthermore, eating more meat mutagens MelQX and DiMelQx, which are produced by cooking over high heat, was associated with disease. In addition, we observed that increased consumption of higher fat lunch meats and liver, along with other meats grilled or barbequed, were associated with aggressive prostate cancer.

Our findings are supported by some previous studies [7], [8], [15] although the general results for overall meat consumption and prostate cancer are certainly mixed. A large prospective cohort study followed 175,343 men for 9 years, during which 10,313 cases of prostate cancer were diagnosed, of which 1,102 were advanced and 419 were fatal [17]. The authors found a significant positive association between increasing consumption of red meat and total prostate cancer and an even stronger association with advanced prostate cancer with approximately 30% higher risk observed in men in the last quintile of intake compared to the first. They saw a trend toward a positive association between red meat consumption and fatal prostate cancer; however, with fewer cases of fatal prostate cancer they were limited in their power to reach statistical significance. Furthermore, the authors found that among the cooking methods they investigated (grilled/barbequed, pan-fried, microwaved and broiled), only meats that were grilled/barbequed showed a significant positive association between meat consumption and prostate cancer. This association was even stronger when looking at men with advanced prostate cancer, with a 36% higher risk of advanced prostate cancer in the highest quintile of meat consumption compared to the lowest. These findings agree with our own, where we observed the association between meat consumption (particularly red meat) and aggressive prostate cancer to be largely driven by grilled or barbequed methods of meat preparation.

Our finding of an association between increased consumption of well or very well done red meat and aggressive prostate cancer is also in agreement with other studies. The Agricultural Health Study identified 668 incident cases of prostate cancer (140 advanced) with 197,017 person years of follow up [22]. The authors found that high intake of well or very well done meat was associated with a 1.26 fold increased risk of incident prostate cancer and a 1.97 fold increased risk of advanced prostate cancer. This is supported by a study of 29,361 men in the Prostate, Lung, Colorectal, and Ovarian Screening Trial, where the authors failed to see an association between overall meat intake and prostate cancer but did see a significant positive association between very well done meat and prostate cancer risk [5].

The mechanism through which the consumption of well-done meat may increase prostate cancer risk is via the release of mutagenic compounds during cooking [30]. Heterocyclic amines (HCAs) and polycyclic aromatic hydrocarbons (PAH) are chemicals formed when muscle meat such as beef, pork, fish or chicken are cooked by high temperature methods such as pan frying or cooking over an open flame [31]. PAHs develop from smoking or grilling meat over an open fire [32]. Fat and juices from cooking meat drip into the fire, causing flames that contain PAHs to coat the surface of the meat. Heterocyclic amines (HCAs) are mutagenic compounds formed during high temperature or long cooking of meat from the reaction of creatine or creatinine, amino acids, and sugar [33]. Both compounds require metabolic activation to carcinogenic intermediates [34]. The HCAs are oxidized and converted to their hydroxyamino derivatives by members of the cytochrome P450 family and further converted to ester forms by acetyltransferase and sulfotransferase. The reactive forms eventually produce DNA adducts through the formation of N-C bonds at guanine bases, resulting in changes in DNA sequences by base substitution, deletion and insertion [35]. The presence of these carcinogen-metabolizing enzymes in the prostate and the relationship between inter-individual variability in these enzymes and prostate cancer risk lend support to their role via carcinogens on this disease [36].

Animal studies have shown that the HCA PhIP can induce the development of tumors in rat prostates [37], [38]. To investigate the association between meat consumption and PhIP levels, Tang et al looked at PhIP-DNA adducts in prostate tumor and adjacent non-tumor cells post radical prostatectomy in 268 men with prostate cancer [27]. They showed that grilled meat consumption was associated with increased PhIP-DNA adduct levels in prostate tumor cells, with red meats and hamburgers displaying the most significant association. While we did not detect a statistically significant association for PhIP, there was a weak trend toward increasing risk. We did, however, find that increasing consumptions of the HCAs MelQx and DiMelQx were associated with an increased risk of aggressive prostate cancer. In support of our findings, the Agricultural Health Study [22] found a borderline significant association between high consumption of the HCAs MelQx and DiMelQx and incident prostate cancer.

The current study has several strengths including its ability to look at various meat types, preparation methods, doneness, and meat mutagens. Furthermore, all cases were men with aggressive prostate cancer, reflecting a disease phenotype that is more likely to progress and require treatment. The study had several limitations. There is a potential for measurement error due to recall bias in the assessment of meat consumption by study participants. The cases were asked to recall their food consumption over the year prior to their diagnosis of prostate cancer, while the controls were asked to recall their food consumption during the previous year. However, since incident cases and controls were recruited into the study at roughly the same time, the period over which recall of dietary intake occurred should be similar between the two groups. Secondly, the food frequency questionnaire had a limited ability to comprehensively assess all the potential food, vitamin and minerals that may effect or confound the associations seen between meat consumption and prostate cancer risk. Futhermore, HCA consumption was deduced using nutrient databases and is therefore subject to the inherent limitations of these databases. Finally, although controls were screened for prostate cancer and evaluated for it if they were thought to be at higher risk of prostate cancer we cannot exclude the potential that controls patients may have prostate cancer that was missed on initial screening and evaluation. However, we would expect the same misclassification of case or control to exist between those with high or low levels of meat consumption. As a result, this misclassification bias would be non-differential and would only attenuate the results. Therefore, the true association between meat consumption and aggressive prostate cancer may be greater if this bias truly exists.

In summary, this study found that high consumption of meats, especially those prepared by grilling, was positively associated with an increased risk of aggressive prostate cancer. Furthermore, increasing intake of well or very well done red meat was positively associated with disease. Although certain mutagenic compounds, such as MelQx and DiMelQx, may play a role in this process, other molecules may also be involved and further studies are required to better characterize the potential role of these compounds in prostate carcinogenesis and to see whether these compounds may be targeted for chemoprevention of prostate cancer.

## Supporting Information

Figure S1 The food frequency questionnaire.
(PDF)
