# Supplementary material for: Impact of Meat Consumption, Preparation, and Mutagens on Aggressive Prostate Cancer
Source: PLoS One. 2011 Nov 23;6(11):e27711. doi: 10.1371/journal.pone.0027711 (PMC3223211; doi:10.1371/journal.pone.0027711)
Supplement: Figure S1 — The food frequency questionnaire. (PDF) [file pone.0027711.s001.pdf]

# Food Questionnaire

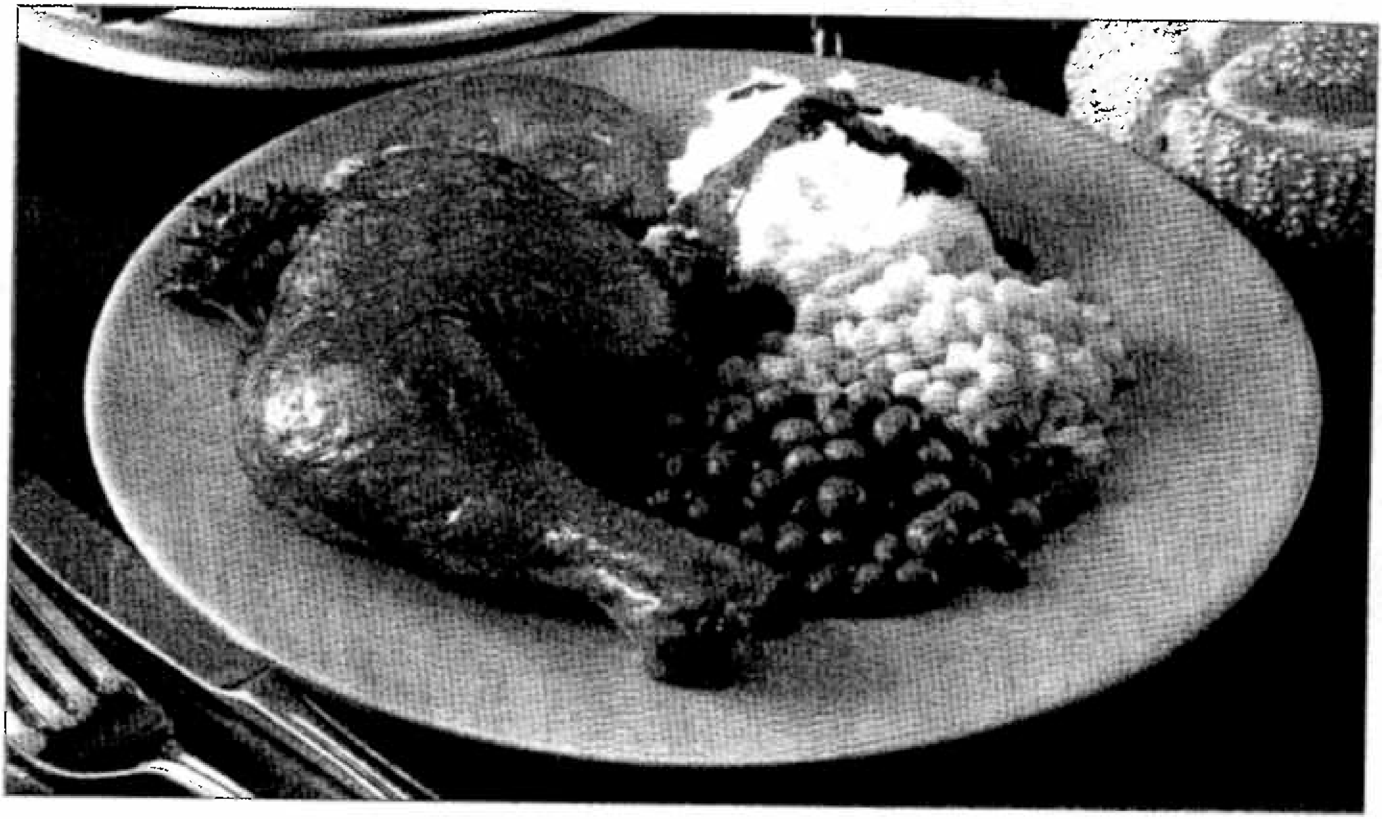

This form asks about your usual food intake during ______________________________.

Please use **pencil**.

Please **print your name** in this box.

Answer by filling in the correct oval.

● Yes ○ No

Do not make any other marks on the form. Please use a separate piece of paper to make comments.

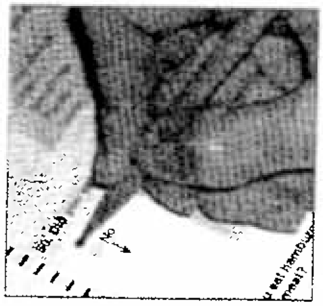

| TODAY'S DATE | | |
|---|---|---|
| MO | DAY | YEAR |

| IDENTIFICATION NUMBER |
|---|

MSEL

PLEASE DO NOT WRITE IN THIS AREA

07518

# [illegible] Food Choices

**These questions are about the types of foods you ate during ________________________________.**

**1. Did you eat chicken or turkey?**

- ○ Yes → **When you ate chicken or turkey, how often did you eat the skin?**
  - ○ Almost always
  - ○ Often
  - ○ Sometimes
  - ○ Rarely
  - ○ Never
- ○ No ↓

**2. Did you eat beef, pork, ham or lamb?**

- ○ Yes → **When you ate beef, pork, ham or lamb, how often did you eat the fat?**
  - ○ Almost always
  - ○ Often
  - ○ Sometimes
  - ○ Rarely
  - ○ Never
- ○ No ↓

**3. Did you eat hamburger or other ground meat?**

- ○ Yes → **When you ate hamburger or other ground meat, was it usually...** *Mark one or two.*
  - ○ Regular
  - ○ Lean
  - ○ Extra lean
  - ○ Ground chicken or turkey
  - ○ Don't know
- ○ No ↓

**4. Did you drink orange, grapefruit or other fruit juices?**

- ○ Yes → **Were any of these vitamins or minerals <u>added</u> (specially fortified) to the juices you drank?** *Mark all that apply.*
  - ○ Extra Vitamin C
  - ○ Vitamin E
  - ○ Calcium
  - ○ None
  - ○ Don't know
- ○ No ↓

**5. Did you eat cold cereals?**

- ○ Yes → **When you ate cold cereal, what type did you usually eat?** *Mark one or two.*
  - ○ Highly fortified cereals (100% of daily values) such as Total®, Smart Start® and Product 19®
  - ○ High fiber or bran cereals such as Raisin Bran® and All Bran®
  - ○ Regular granola (not lowfat)
  - ○ All other cereals such as lowfat granola, Cheerios®, Corn Flakes® and Frosted Flakes®
- ○ No ↓

**6. Did you put milk (all types), cream or creamer on cereal?**

- ○ Yes → **When you put milk, cream or creamer on cereal, what type did you usually use?** *Mark one or two.*
  - ○ Cream or half and half
  - ○ Whole milk
  - ○ 2% milk
  - ○ 1% milk or buttermilk
  - ○ Nonfat or skim milk
  - ○ Soy milk
  - ○ Non-dairy creamer
  - ○ Don't know
- ○ No ↓

**7. Did you put milk (all types), cream or creamer in coffee or tea?**

- ○ Yes → **When you put milk, cream or creamer in coffee or tea, what type did you usually use?** *Mark one or two.*
  - ○ Cream or half and half
  - ○ Whole milk
  - ○ 2% milk
  - ○ 1% milk or buttermilk
  - ○ Nonfat or skim milk
  - ○ Soy milk
  - ○ Non-dairy creamer
  - ○ Don't know
- ○ No ↓

**8. Did you drink milk (all types)?** *Also include beverages made with milk, such as lattes, cappuccinos, mochas or hot chocolate.*

- ○ Yes → **When you drank milk or beverages made with milk, was it usually...** *Mark one or two.*
  - ○ Whole milk
  - ○ 2% milk
  - ○ 1% milk or buttermilk
  - ○ Nonfat or skim milk
  - ○ Soy milk
  - ○ Don't know
- ○ No ↓

**9. Did you use salad dressing?**

- ○ Yes → **When you used salad dressing, what type did you usually use?** *Mark one or two.*
  - ○ Regular, including oil and vinegar
  - ○ Low or reduced fat
  - ○ Fat free or nonfat
- ○ No ↓

**10. Did you use mayonnaise?**

- ○ Yes → **When you used mayonnaise, what type did you usually use?** *Mark one or two.*
  - ○ Regular
  - ○ Low or reduced fat
  - ○ Fat free or nonfat
- ○ No ↓

**11. Did you eat cookies or cakes?**

- ○ Yes → **When you ate cookies or cakes, how often were they fig bars, SnackWell's®, angel food cakes, or other types of low or nonfat cookies or cakes?**
  - ○ Almost always
  - ○ Often
  - ○ Sometimes
  - ○ Rarely
  - ○ Never
- ○ No ↓

**12. In your household, what kinds of fat were usually used when cooking, for example to flavor vegetables or fry meat?** *Mark up to four.*

- ○ Butter
- ○ Stick margarine
- ○ Tub or liquid margarine
- ○ Lowfat margarine
- ○ Olive oil
- ○ Canola oil
- ○ Other oils such as corn, soybean, peanut and safflower
- ○ Lard, bacon fat or meat drippings
- ○ Didn't use fat or used non-stick spray (Pam®)

**13. What kinds of fat did you use at the table, for example on breads, vegetables or potatoes?** *Mark up to four.*

- ○ Butter
- ○ Stick margarine
- ○ Tub or liquid margarine
- ○ Lowfat margarine
- ○ Olive oil
- ○ Sour cream
- ○ Didn't use fat

PLEASE DO NOT WRITE IN THIS AREA

07518

These questions are about foods you ate during ______________________________.

**14. Mark the column to show how often, on average, you ate the following foods. Mark your usual serving size as small, medium or large.**

- A small serving is about one-half (1/2) the medium serving size or less.
- A large serving is about one-and-a-half (1 1/2) times the medium serving size or more.

**EXAMPLE:** This man ate spaghetti with meat sauce every Saturday. He usually ate about 2 1/2 cups.

| | HOW OFTEN DID YOU EAT THESE FOODS? → | | | | | | | | AMOUNT? | | | |
|---|---|---|---|---|---|---|---|---|---|---|---|---|
| | NEVER or less than once per month | 1 per month | 2-3 per month | 1 per week | 2 per week | 3-4 per week | 5-6 per week | 1 per day | 2+ per day | Medium serving size | S | M | L |
| Spaghetti, lasagna, and other pasta with tomato with meat sauce | ○ | ○ | ○ | ● | ○ | ○ | ○ | ○ | ○ | 1 1/2 cups | ○ | ○ | ● |

## CEREALS, BREADS, SNACKS

| | HOW OFTEN DID YOU EAT THESE FOODS? → | | | | | | | | AMOUNT? | | | |
|---|---|---|---|---|---|---|---|---|---|---|---|---|
| | NEVER or less than once per month | 1 per month | 2-3 per month | 1 per week | 2 per week | 3-4 per week | 5-6 per week | 1 per day | 2+ per day | Medium serving size | S | M | L |
| Cold cereals | ○ | ○ | ○ | ○ | ○ | ○ | ○ | ○ | ○ | 1 1/2 cups | ○ | ○ | ○ |
| Cooked cereals and grits | ○ | ○ | ○ | ○ | ○ | ○ | ○ | ○ | ○ | 1 1/2 cups | ○ | ○ | ○ |
| Milk on cereals | ○ | ○ | ○ | ○ | ○ | ○ | ○ | ○ | ○ | 3/4 cup | ○ | ○ | ○ |
| Pancakes, French toast and waffles | ○ | ○ | ○ | ○ | ○ | ○ | ○ | ○ | ○ | 2 pieces | ○ | ○ | ○ |
| Muffins, scones, croissants and biscuits | ○ | ○ | ○ | ○ | ○ | ○ | ○ | ○ | ○ | 1 medium | ○ | ○ | ○ |
| White breads, including bagels, rolls and English muffins | ○ | ○ | ○ | ○ | ○ | ○ | ○ | ○ | ○ | 2 slices or 1 medium | ○ | ○ | ○ |
| Dark breads, including dark bagels and rolls | ○ | ○ | ○ | ○ | ○ | ○ | ○ | ○ | ○ | 2 slices or 1 medium | ○ | ○ | ○ |
| Cornbread and corn muffins | ○ | ○ | ○ | ○ | ○ | ○ | ○ | ○ | ○ | 2 slices or 1 medium | ○ | ○ | ○ |
| Butter or margarine on breads, cereals, pancakes, etc. | ○ | ○ | ○ | ○ | ○ | ○ | ○ | ○ | ○ | 2 pats or 2 teaspoons | ○ | ○ | ○ |
| Jam, jelly, honey and syrup | ○ | ○ | ○ | ○ | ○ | ○ | ○ | ○ | ○ | 2 Tbsp. | ○ | ○ | ○ |
| Granola bars and cereal bars such as Nutri-Grain Bars® | ○ | ○ | ○ | ○ | ○ | ○ | ○ | ○ | ○ | 1 bar | ○ | ○ | ○ |
| Sports or meal replacement bars such as Power Bars® and Clif Bars® | ○ | ○ | ○ | ○ | ○ | ○ | ○ | ○ | ○ | 1 bar | ○ | ○ | ○ |

## CEREALS, BREADS, SNACKS (continued)

HOW OFTEN DID YOU EAT THESE FOODS? → AMOUNT?

| | NEVER or less than once per month | 1 per month | 2-3 per month | 1 per week | 2 per week | 3-4 per week | 5-6 per week | 1 per day | 2+ per day | Medium serving size | S | M | L |
|---|---|---|---|---|---|---|---|---|---|---|---|---|---|
| Low or nonfat potato chips, tortilla chips and corn chips | | | | | | | | | | 4 handfuls or 1 med.bag | | | |
| Regular potato chips, tortilla chips, corn chips and puffs | | | | | | | | | | 4 handfuls or 1 med.bag | | | |
| Plain popcorn (no butter) or lowfat microwave popcorn | | | | | | | | | | 8 handfuls | | | |
| Buttered or regular microwave popcorn | | | | | | | | | | 8 handfuls | | | |
| Low or nonfat crackers, such as saltines and SnackWell's® | | | | | | | | | | 12 medium | | | |
| Regular crackers, such as Ritz® and Wheat Thins® | | | | | | | | | | 12 medium | | | |
| Peanut butter, peanuts and other nuts and seeds | | | | | | | | | | 2 Tbsp. (spreads) or ½ cup (nuts) | | | |

## MEAT, FISH, EGGS

HOW OFTEN DID YOU EAT THESE FOODS? → AMOUNT?

| | NEVER or less than once per month | 1 per month | 2-3 per month | 1 per week | 2 per week | 3-4 per week | 5-6 per week | 1 per day | 2+ per day | Medium serving size | S | M | L |
|---|---|---|---|---|---|---|---|---|---|---|---|---|---|
| Eggs | | | | | | | | | | 2 eggs | | | |
| Bacon and breakfast sausage | | | | | | | | | | 3 strips or 2 links | | | |
| Low or reduced fat hot dogs and sausage | | | | | | | | | | 2 hot dogs or 4 ounces | | | |
| Regular hot dogs and sausage such as bratwurst and chorizo | | | | | | | | | | 2 hot dogs or 4 ounces | | | |
| Lunch meats such as ham, turkey and lowfat bologna | | | | | | | | | | 2 slices | | | |
| All other lunch meat such as bologna, salami and Spam® | | | | | | | | | | 2 slices | | | |
| Canned tuna, tuna salad and tuna casserole | | | | | | | | | | 1 can tuna or 2 cups casserole | | | |
| Beef, pork, ham and lamb | | | | | | | | | | 6 ounces | | | |
| Ground meat, including hamburgers and meatloaf | | | | | | | | | | 1 medium or 4 ounces | | | |
| Liver, chicken liver and organ meats | | | | | | | | | | 6 ounces | | | |
| Fried chicken, including nuggets and tenders | | | | | | | | | | 2 large pieces or 9 nuggets | | | |

PLEAS[illegible]

## MEAT, FISH, EGGS (continued)

| | HOW OFTEN DID YOU EAT THESE FOODS? | | | | | | | | | → AMOUNT? | | | |
|---|---|---|---|---|---|---|---|---|---|---|---|---|---|
| | NEVER or less than once per month | 1 per month | 2-3 per month | 1 per week | 2 per week | 3-4 per week | 5-6 per week | 1 per day | 2+ per day | Medium serving size | S | M | L |
| Chicken and turkey (roasted, stewed, grilled or broiled) | | | | | | | | | | 2 large or 4 small pieces | | | |
| Fried fish, fish sandwich and fried shellfish (shrimp and oysters) | | | | | | | | | | 4 ounces or 1 sandwich | | | |
| Shellfish, not fried (shrimp, lobster, crab and oysters) | | | | | | | | | | 4 ounces or 1 cup | | | |
| White fish (broiled or baked) such as sole, halibut, snapper and cod | | | | | | | | | | 6 ounces | | | |
| Dark fish (broiled or baked) such as salmon, mackerel and bluefish | | | | | | | | | | 6 ounces | | | |

## SPAGHETTI, MIXED DISHES, SOUPS

| | HOW OFTEN DID YOU EAT THESE FOODS? | | | | | | | | | → AMOUNT? | | | |
|---|---|---|---|---|---|---|---|---|---|---|---|---|---|
| | NEVER or less than once per month | 1 per month | 2-3 per month | 1 per week | 2 per week | 3-4 per week | 5-6 per week | 1 per day | 2+ per day | Medium serving size | S | M | L |
| Stew, pot pie, curries and casseroles with meat or chicken | | | | | | | | | | 1½ cups | | | |
| Chili with meat and beans | | | | | | | | | | 1½ cups | | | |
| Spaghetti, lasagna and other pasta with tomato with meat sauce | | | | | | | | | | 1½ cups | | | |
| Spaghetti and other pasta with tomato sauce (no meat) | | | | | | | | | | 1½ cups | | | |
| Spaghetti and other pasta with oil, cheese or cream sauce, including macaroni and cheese | | | | | | | | | | 1½ cups | | | |
| Asian-style (stir-fried) noodles and rice, such as chow mein, fried rice and Pad Thai | | | | | | | | | | 1½ cups | | | |
| Pizza | | | | | | | | | | 3 slices | | | |
| Tofu, tempeh and products such as tofu hot dogs, soy burgers and tofu cheese | | | | | | | | | | 4 ounces, 2 hot dogs or 1 burger | | | |
| Burritos, tacos, tostadas and quesadillas | | | | | | | | | | 2 medium | | | |
| Enchiladas and tamales | | | | | | | | | | 2 medium | | | |
| Vegetable, minestrone and tomato soup | | | | | | | | | | 1½ cups | | | |
| Cream soups such as chowders, potato and cheese | | | | | | | | | | 1½ cups | | | |

## SPAGHETTI, MIXED DISHES, SOUPS (continued)

| | HOW OFTEN DID YOU EAT THESE FOODS? | | | | | | | | → | AMOUNT? | | |
|---|---|---|---|---|---|---|---|---|---|---|---|---|
| | NEVER or less than once per month | 1 per month | 2-3 per month | 1 per week | 2 per week | 3-4 per week | 5-6 per week | 1 per day | 2+ per day | Medium serving size | S | M | L |
| Bean soups such as pea, lentil and black bean | | | | | | | | | | 1½ cups | | | |
| Miso soup | | | | | | | | | | 1½ cups | | | |
| Ramen noodle soup | | | | | | | | | | 1½ cups | | | |
| Other soups such as chicken noodle | | | | | | | | | | 1½ cups | | | |

## DAIRY PRODUCTS

| | HOW OFTEN DID YOU EAT THESE FOODS? | | | | | | | | → | AMOUNT? | | |
|---|---|---|---|---|---|---|---|---|---|---|---|---|
| | NEVER or less than once per month | 1 per month | 2-3 per month | 1 per week | 2 per week | 3-4 per week | 5-6 per week | 1 per day | 2+ per day | Medium serving size | S | M | L |
| Cottage cheese and ricotta cheese | | | | | | | | | | ¾ cup | | | |
| Low or reduced fat cheese, including cheese used in cooking | | | | | | | | | | 2 slices or ½ cup shredded | | | |
| All other cheese (American, cheddar or cream), including cheese used in cooking | | | | | | | | | | 2 slices, ½ cup shredded or 2 Tbsp. cream | | | |
| Yogurt, all types except frozen | | | | | | | | | | 1 cup | | | |

## VEGETABLES and GRAINS

| | HOW OFTEN DID YOU EAT THESE FOODS? | | | | | | | | → | AMOUNT? | | |
|---|---|---|---|---|---|---|---|---|---|---|---|---|
| | NEVER or less than once per month | 1 per month | 2-3 per month | 1 per week | 2 per week | 3-4 per week | 5-6 per week | 1 per day | 2+ per day | Medium serving size | S | M | L |
| ***Mark all vegetables you ate, including in salads, mixed dishes, sandwiches and stir-fries.*** | | | | | | | | | | | | | |
| Green salad (lettuce or spinach) | | | | | | | | | | 2 cups | | | |
| Salad dressing (all types) | | | | | | | | | | ¼ cup | | | |
| Fresh tomatoes | | | | | | | | | | 1 medium or 4 slices | | | |
| Carrots | | | | | | | | | | ¾ cup | | | |
| Green peppers and green chilies | | | | | | | | | | ½ cup | | | |
| Red peppers and red chilies | | | | | | | | | | ½ cup | | | |

## VEGETABLES and GRAINS (continued)

| | HOW OFTEN DID YOU EAT THESE FOODS? | | | | | | | | | → | AMOUNT? | | |
|---|---|---|---|---|---|---|---|---|---|---|---|---|---|
| | NEVER or less than once per month | 1 per month | 2-3 per month | 1 per week | 2 per week | 3-4 per week | 5-6 per week | 1 per day | 2+ per day | Medium serving size | S | M | L |
| ***Mark all vegetables you ate, including in salads, mixed dishes, sandwiches and stir-fries.*** | | | | | | | | | | | | | |
| Broccoli | | | | | | | | | | 3/4 cup | | | |
| Cauliflower, cabbage and Brussels sprouts | | | | | | | | | | 3/4 cup | | | |
| Green or string beans | | | | | | | | | | 3/4 cup | | | |
| Green peas | | | | | | | | | | 3/4 cup | | | |
| Corn and hominy | | | | | | | | | | 3/4 cup | | | |
| Summer squash and zucchini | | | | | | | | | | 3/4 cup | | | |
| Winter squash such as acorn, butternut and pumpkin | | | | | | | | | | 3/4 cup | | | |
| Yams and sweet potatoes | | | | | | | | | | 1 medium | | | |
| Cooked greens such as spinach, mustard greens and collards | | | | | | | | | | 3/4 cup | | | |
| Onions and leeks | | | | | | | | | | 1/2 cup | | | |
| Fresh garlic, including in cooking | | | | | | | | | | 1 clove | | | |
| Avocado and guacamole | | | | | | | | | | 1/2 medium or 1/2 cup | | | |
| French fries, fried potatoes and hash browns | | | | | | | | | | 1 cup | | | |
| Potatoes (boiled, baked or mashed) | | | | | | | | | | 1 medium or 1 cup | | | |
| Refried beans | | | | | | | | | | 3/4 cup | | | |
| All other beans (baked, lima or chili without meat) | | | | | | | | | | 3/4 cup | | | |
| Coleslaw | | | | | | | | | | 3/4 cup | | | |
| Potato, macaroni and pasta salads made with mayonnaise or oil | | | | | | | | | | 3/4 cup | | | |
| Rice, noodles and other grains (as a side dish) | | | | | | | | | | 3/4 cup | | | |
| Butter, margarine, sour cream and other fat added to vegetables, potatoes and rice | | | | | | | | | | 2 pats or 2 teaspoons | | | |

PLEASE [illegible]

07518

## SAUCES and CONDIMENTS

| | HOW OFTEN DID YOU EAT THESE FOODS? | | | | | | | | | → | AMOUNT? | | |
|---|---|---|---|---|---|---|---|---|---|---|---|---|---|
| | NEVER or less than once per month | 1 per month | 2-3 per month | 1 per week | 2 per week | 3-4 per week | 5-6 per week | 1 per day | 2+ per day | Medium serving size | S | M | L |
| Cheese sauce and cream sauce | | | | | | | | | | ½ cup | | | |
| Meat gravies | | | | | | | | | | ½ cup | | | |
| Ketchup | | | | | | | | | | ¼ cup | | | |
| Salsa (as dip or on foods) | | | | | | | | | | ½ cup | | | |
| Mayonnaise and mayonnaise-type spreads | | | | | | | | | | 2 Tbsp. | | | |

## FRUITS

| | HOW OFTEN DID YOU EAT THESE FOODS? | | | | | | | | | → | AMOUNT? | | |
|---|---|---|---|---|---|---|---|---|---|---|---|---|---|
| | NEVER or less than once per month | 1 per month | 2-3 per month | 1 per week | 2 per week | 3-4 per week | 5-6 per week | 1 per day | 2+ per day | Medium serving size | S | M | L |
| Apples, applesauce and pears | | | | | | | | | | 1 medium or ½ cup | | | |
| Bananas | | | | | | | | | | 1 medium | | | |
| Peaches, nectarines and plums | | | | | | | | | | 1 medium or ½ cup | | | |
| Apricots (fresh, canned or dried) | | | | | | | | | | 2 medium or 4 halves | | | |
| Dried fruit (other than apricots) such as raisins and prunes | | | | | | | | | | ½ cup | | | |
| Oranges, grapefruit and tangerines (not juice) | | | | | | | | | | 1 orange or ½ grapefruit | | | |
| Berries such as strawberries and blueberries | | | | | | | | | | ¾ cup | | | |
| Cantaloupe, orange melon and mango (in season) | | | | | | | | | | ¼ melon or ½ mango | | | |
| Watermelon and red melon | | | | | | | | | | 1 large slice | | | |
| Any other fruit such as grapes, fruit cocktail, pineapple and cherries | | | | | | | | | | ¾ cup | | | |

## SWEETS

| | HOW OFTEN DID YOU EAT THESE FOODS? | | | | | | | | | → | AMOUNT? | | |
|---|---|---|---|---|---|---|---|---|---|---|---|---|---|
| | NEVER or less than once per month | 1 per month | 2-3 per month | 1 per week | 2 per week | 3-4 per week | 5-6 per week | 1 per day | 2+ per day | Medium serving size | S | M | L |
| Low or nonfat frozen desserts such as lowfat ice cream, frozen yogurt and sherbet | ○ | ○ | ○ | ○ | ○ | ○ | ○ | ○ | ○ | 2 scoops | ○ | ○ | ○ |
| Ice cream and milkshakes | ○ | ○ | ○ | ○ | ○ | ○ | ○ | ○ | ○ | 2 scoops or 1 shake | ○ | ○ | ○ |
| Pudding, custard and flan | ○ | ○ | ○ | ○ | ○ | ○ | ○ | ○ | ○ | 1 cup | ○ | ○ | ○ |
| Doughnuts, pies and pastries | ○ | ○ | ○ | ○ | ○ | ○ | ○ | ○ | ○ | 1 piece | ○ | ○ | ○ |
| Cookies and cakes | ○ | ○ | ○ | ○ | ○ | ○ | ○ | ○ | ○ | 3 med. cookies or 1 piece of cake | ○ | ○ | ○ |
| Chocolate, candy bars and toffee | ○ | ○ | ○ | ○ | ○ | ○ | ○ | ○ | ○ | 1 regular bar or 4 pieces | ○ | ○ | ○ |
| Other candy, such as Lifesavers®, licorice and jelly beans | ○ | ○ | ○ | ○ | ○ | ○ | ○ | ○ | ○ | 6 pieces or 25 jelly beans | ○ | ○ | ○ |

## PLEASE ANSWER THESE THREE IMPORTANT QUESTIONS!

***Note that the frequency headings are different.***

| | NEVER or less than once per week | 1-2 per week | 3-4 per week | 5-6 per week | 1 per day | 2 per day | 3 per day | 4 per day | 5+ per day |
|---|---|---|---|---|---|---|---|---|---|
| How often did you eat foods that were cooked in fat (pan-fried, sautéed, or deep-fried)? *Count all fat such as margarine, butter, oil or lard.* | ○ | ○ | ○ | ○ | ○ | ○ | ○ | ○ | ○ |
| How often did you eat a serving of vegetables? *Do not count potatoes, salad or beans.* | ○ | ○ | ○ | ○ | ○ | ○ | ○ | ○ | ○ |
| How often did you eat a serving of fruit? *Do not count juices.* | ○ | ○ | ○ | ○ | ○ | ○ | ○ | ○ | ○ |

PLEASE DO NOT WRITE IN THIS AREA

07518

## BEVERAGES and ALCOHOL

HOW OFTEN DID YOU DRINK THESE BEVERAGES? → AMOUNT?

***Note that the frequency headings are different.***

| | NEVER or less than once per month | 1-3 per month | 1 per week | 2-4 per week | 5-6 per week | 1 per day | 2-3 per day | 5-6 per day | 6+ per day | Medium serving size | S | M | L |
|---|---|---|---|---|---|---|---|---|---|---|---|---|---|
| Milk (all types) as a beverage | | | | | | | | | | 1 cup | | | |
| Latte, cappuccino, mocha or hot chocolate | | | | | | | | | | 1 cup | | | |
| Coffee (not lattes or mochas) | | | | | | | | | | 1 cup | | | |
| Tea (all types) | | | | | | | | | | 1 cup | | | |
| Milk, cream or creamer added to tea and coffee | | | | | | | | | | 1 Tbsp. | | | |
| Tomato juice, V-8® and other vegetable juices | | | | | | | | | | 1 cup | | | |
| Orange juice and grapefruit juice | | | | | | | | | | 1 cup | | | |
| Other 100% fruit juice, such as apple, grape and cranberry | | | | | | | | | | 1 cup | | | |
| Fruit drinks fortified with Vitamin C, such as Hi-C®, Fruitopia® and Kool-Aid® | | | | | | | | | | 1 cup | | | |
| Meal replacement drinks and shakes such as Slim-Fast®, Ensure® and Carnation Instant Breakfast® | | | | | | | | | | 1 cup | | | |
| Diet soft drinks | | | | | | | | | | 12 ounces or 1 can | | | |
| Regular soft drinks | | | | | | | | | | 12 ounces or 1 can | | | |
| Water (tap, bottled or sparkling) | | | | | | | | | | 1 cup | | | |
| Beer (all types) | | | | | | | | | | 12 ounce can or bottle | | | |
| Red wine | | | | | | | | | | 1 medium glass (6 oz) | | | |
| White or rosé wine | | | | | | | | | | 1 medium glass (6 oz) | | | |
| Liquor and mixed drinks | | | | | | | | | | 1 shot (1½oz) or 1 mixed drink | | | |

# THANK YOU!

**Please take a moment to fill in any questions you may have skipped.**

# Supplement Use Questionnaire

## MULTIVITAMINS

**MULTIVITAMINS** contain 10 or more vitamins and/or minerals. Examples include One-A-Day® multivitamins, Centrum®, and vitamin packs containing several pills.

**1. In the past 10 years, have you taken a MULTIVITAMIN <u>at least once a week for a year</u>?**

- o No → **Skip to Question 7, middle of Page 3**
- o Yes, at least once a week for a year

↓

**a) Years taken in past 10 years**
o 1-3 o 4-6 o 7-9 o 10

**b) Days per week**
o 1-2 o 3-4 o 5-6 o 7

**2. Did you take a MULTIVITAMIN <u>in the last 3 months</u>?**

- o No → **Skip to Question 7, middle of Page 3**
- o Yes

**3. What brand of MULTIVITAMIN did you take in the last 3 months?**
*Mark only one.*

- o Centrum®
- o Centrum Silver®
- o Kirkland®
- o Kirkland® Premium with herbals
- o NatureMade® with minerals
- o NatureMade® (no minerals)
- o One-A-Day® Essential
- o One-A-Day® Maximum
- o One-A-Day® Men's
- o One-A-Day® 50 Plus
- o Theragran-M® with minerals
- o Theragran® (no minerals)
- o Unicap® Plus Iron
- o Unicap® M
- o Unicap® Senior

→ **How long have you taken this brand of MULTIVITAMIN?**
o 1-3 years o 7-9 years
o 4-6 years o 10 years or more

**Skip to Question 5, top of Page 3**

- o **My brand is not listed above** → **Continue to Question 4, next page**

4. **What brand of MULTIVITAMIN did you take in the last three months?**________________________

*Please look at the label and tell us what is in the MULTIVITAMIN. The most common amounts are listed. If your multivitamin contains a different amount, choose the range that includes the amount you take.*

| | Amount per day | (or in this range) |
|---|---|---|
| **Total Vitamin A in Multivitamin** o Yes → o No | o 5000 IU | (1-5999 IU) |
| | o 7500 IU | (6000-8999 IU) |
| | o 10,000 IU | (9000-12,999 IU) |
| | o 15,000 IU | (13,000+ IU) |

**Is Beta-carotene included?** o Yes o No

| | Amount per day | (or in this range) |
|---|---|---|
| **Vitamin C in Multivitamin** o Yes → o No | o 60 mg | (1-74 mg) |
| | o 100 mg | (75-199 mg) |
| | o 250 mg | (200-399 mg) |
| | o 500 mg | (400-799 mg) |
| | o 1000 mg | (800+ mg) |
| **Vitamin B6 in Multivitamin** o Yes → o No | o 2 mg | (1-9 mg) |
| | o 20 mg | (10-39 mg) |
| | o 50 mg | (40-74 mg) |
| | o 100 mg | (75+ mg) |
| **Folic acid (folate) in Multivitamin** o Yes → o No | o 400 mcg | (1-499 mcg) |
| | o 600 mcg | (500-699 mcg) |
| | o 800 mcg | (700+ mcg) |
| **Vitamin B12 in Multivitamin** o Yes → o No | o 6 mcg | (1-39 mcg) |
| | o 50 mcg | (40-74 mcg) |
| | o 100 mcg | (75-199 mcg) |
| | o 250 mcg | (200+ mcg) |
| **Vitamin E in Multivitamin** o Yes → o No | o 30 IU | (1-74 IU) |
| | o 100 IU | (75-149 IU) |
| | o 200 IU | (150-299 IU) |
| | o 400 IU | (300-499 IU) |
| | o 600 IU | (500+ IU) |

| | Amount per day | (or in this range) |
|---|---|---|
| **Calcium in Multivitamin** o Yes → o No | o 100 mg | (1-199 mg) |
| | o 250 mg | (200-399 mg) |
| | o 500 mg | (400-699 mg) |
| | o 800 mg | (700-899 mg) |
| | o 1000 mg | (900+ mg) |
| **Zinc in Multivitamin** o Yes → o No | o 15 mg | (1-19 mg) |
| | o 30 mg | (20-49 mg) |
| | o 60 mg | (50-74 mg) |
| | o 100 mg | (75+ mg) |
| **Selenium in Multivitamin** o Yes → o No | o 25 mcg | (1-39 mcg) |
| | o 50 mcg | (40-74 mcg) |
| | o 100 mcg | (75+ mcg) |

**Does your MULTIVITAMIN contain any of these other vitamins and minerals?**
*Mark all that apply.*

- o Thiamin (B1)
- o Riboflavin (B2)
- o Niacin (B3)
- o Vitamin D
- o Magnesium
- o Copper
- o Chromium
- o Iron

**Does your MULTIVITAMIN contain any of these other compounds?** *Mark all that apply.*

- o Ginseng
- o Bioflavonoids
- o Lycopene
- o Dong quai
- o Saw palmetto
- o Garlic
- o Soy or isoflavones
- o Lutein

**How long have you taken this brand of MULTIVITAMIN?**

- o 1-3 years
- o 4-6 years
- o 7-9 years
- o 10 years



**Turn Over**

5. **In the past 10 years, did you take a different brand of MULTIVITAMIN?**

- o No → Skip to Question 7
- o Yes

6. **In the past 10 years, what brand of MULTIVITAMIN did you take most often?**

| | |
|---|---|
| o Centrum® | o Theragran-M® (minerals) |
| o Centrum Silver® | o Unicap® Plus Iron |
| o NatureMade® with minerals | o Unicap® M |
| o NatureMade® (no minerals) | o Unicap® Senior |
| o One-A-Day® with minerals | o Generic or store brand |
| o One-A-Day® (no minerals) | o Other brand(s) |
| o Theragran® (no minerals) | o Don't know |

## VITAMINS, MINERALS AND OTHER SUPPLEMENTS (not including multivitamins)

7. **In the past 10 years, have you taken any dietary supplements (other than a multivitamin) for at least a year?**

*Include vitamins, minerals, herbals and mixtures. Also include calcium, Tums® and other antacid tablets that contain calcium.*

- o No → Skip to Question 9, bottom of Page 6
- o Yes, but less than once a week → Skip to Question 9, bottom of Page 6
- o Yes, at least once a week for a year

8. **In the past 10 years, which vitamins, minerals, and herbals are (or were) in your supplements? Do NOT include multivitamins.**

*If you have the bottles, please look at the labels. The most common amounts are listed. If your supplement contains a different amount, choose the range that includes the amount you take.*

**Vitamin D**

- o Take now / o Took only in past
  - **a) Years taken in past 10 years**: o 1-3 o 4-6 o 7-9 o 10
  - **b) Days per week**: o 1-2 o 3-4 o 5-6 o 7
  - **c) Amount per day (or in this range)**
    - o 400 IU (1-499 IU)
    - o 600 IU (500-699 IU)
    - o 800 IU (700+ IU)
    - o Don't know
- o Never taken

**Folic acid (folate)**

- o Take now / o Took only in past
  - **a) Years taken in past 10 years**: o 1-3 o 4-6 o 7-9 o 10
  - **b) Days per week**: o 1-2 o 3-4 o 5-6 o 7
  - **c) Amount per day (or in this range)**
    - o 400 mcg (1-499 mcg)
    - o 600 mcg (500-699 mcg)
    - o 800 mcg (700+ mcg)
    - o Don't know
- o Never taken



Turn Over

**Vitamin A**

- o Take now
- o Took only in past

a) Years taken in past 10 years
o 1-3 o 4-6 o 7-9 o 10

b) Days per week
o 1-2 o 3-4 o 5-6 o 7

c) Amount per day (or in this range)
- o 5000 IU (1-5999 IU)
- o 7500 IU (6-8999 IU)
- o 10,000 IU (9-12,999 IU)
- o 15,000 IU (13-17,999 IU)
- o 20,000 IU (18,000+ IU)
- o Don't know

o Never taken

**Beta-carotene**

- o Take now
- o Took only in past

a) Years taken in past 10 years
o 1-3 o 4-6 o 7-9 o 10

b) Days per week
o 1-2 o 3-4 o 5-6 o 7

c) Amount per day (or in this range)
- o 5000 IU (1-5999 IU)
- o 7500 IU (6-8999 IU)
- o 10,000 IU (9-12,999 IU)
- o 15,000 IU (13-17,999 IU)
- o 20,000 IU (18,000+ IU)
- o Don't know

o Never taken

**Vitamin C**

- o Take now
- o Took only in past

a) Years taken in past 10 years
o 1-3 o 4-6 o 7-9 o 10

b) Days per week
o 1-2 o 3-4 o 5-6 o 7

c) Amount per day (or in this range)
- o 60 mg (1-74 mg)
- o 100 mg (75-199 mg)
- o 250 mg (200-399 mg)
- o 500 mg (400-799 mg)
- o 1000 mg (800-1299 mg)
- o 1500 mg (1300+ mg)
- o Don't know

o Never taken

**Vitamin E**

- o Take now
- o Took only in past

a) Years taken in past 10 years
o 1-3 o 4-6 o 7-9 o 10

b) Days per week
o 1-2 o 3-4 o 5-6 o 7

c) Amount per day (or in this range)
- o 30 IU (1-74 IU)
- o 100 IU (75-149 IU)
- o 200 IU (150-299 IU)
- o 400 IU (300-499 IU)
- o 600 IU (500-699 IU)
- o 800 IU (700+ IU)
- o Don't know

o Never taken

**Niacin (B3) or nicotinic acid**

- o Take now
- o Took only in past

a) Years taken in past 10 years
o 1-3 o 4-6 o 7-9 o 10

b) Days per week
o 1-2 o 3-4 o 5-6 o 7

c) Amount per day (or in this range)
- o 20 mg (1-39 mg)
- o 50 mg (40-74 mg)
- o 100 mg (75-199 mg)
- o 250 mg (200-999 mg)
- o 1000 mg (1g) (1g or more)
- o Don't know

o Never taken

**Vitamin B6**

- o Take now
- o Took only in past

a) Years taken in past 10 years
o 1-3 o 4-6 o 7-9 o 10

b) Days per week
o 1-2 o 3-4 o 5-6 o 7

c) Amount per day (or in this range)
- o 2 mg (1-14 mg)
- o 50 mg (15-74 mg)
- o 100 mg (75-199 mg)
- o 250 mg (200+ mg)
- o Don't know

o Never taken



**Turn Over**

## Vitamin B12

o Take now
o Took only in past

a) Years taken in past 10 years
o 1-3 o 4-6 o 7-9 o 10

b) Days per week
o 1-2 o 3-4 o 5-6 o 7

c) Amount per day (or in this range)
o 6 mcg (1-39 mcg)
o 50 mcg (40-74 mcg)
o 100 mcg (75-199 mcg)
o 250 mcg (200-399 mcg)
o 500 mcg (400+ mcg)
o Don't know

o Never taken

## Calcium, Tums®, or antacids with calcium*

*Regular tablets = 200 mg per tablet
Ultra or maximum tablets = 400 mg per tablet

o Take now
o Took only in past

a) Years taken in past 10 years
o 1-3 o 4-6 o 7-9 o 10

b) Days per week
o 1-2 o 3-4 o 5-6 o 7

c) Amount per day (or in this range)
o 100 mg (1-199 mg)
o 250 mg (200-399 mg)
o 500 mg (400-699 mg)
o 800 mg (700-899 mg)
o 1000 mg (900-1299 mg)
o 1500 mg (1300+ mg)
o Don't know

o Never taken

## Iron

o Take now
o Took only in past

a) Years taken in past 10 years
o 1-3 o 4-6 o 7-9 o 10

b) Days per week
o 1-2 o 3-4 o 5-6 o 7

c) Amount per day (or in this range)
o 10 mg (1-14 mg)
o 18 mg (15-24 mg)
o 27 mg (25-34 mg)
o 50 mg (35+ mg)
o Don't know

o Never taken

## Zinc

o Take now
o Took only in past

a) Years taken in past 10 years
o 1-3 o 4-6 o 7-9 o 10

b) Days per week
o 1-2 o 3-4 o 5-6 o 7

c) Amount per day (or in this range)
o 15 mg (1-19 mg)
o 30 mg (20-49 mg)
o 60 mg (50-74 mg)
o 100 mg (75+ mg)
o Don't know

o Never taken

## Selenium

o Take now
o Took only in past

a) Years taken in past 10 years
o 1-3 o 4-6 o 7-9 o 10

b) Days per week
o 1-2 o 3-4 o 5-6 o 7

c) Amount per day (or in this range)
o 25 mcg (1-39 mcg)
o 50 mcg (40-74 mcg)
o 100 mcg (75-149 mcg)
o 200 mcg (150+ mcg)
o Don't know

o Never taken

## Lycopene

o Take now
o Took only in past

a) Years taken in past 10 years
o 1-3 o 4-6 o 7-9 o 10

b) Days per week
o 1-2 o 3-4 o 5-6 o 7

c) Amount per day (or in this range)
o 5 mg (1-7 mg)
o 10 mg (8-12 mg)
o 15 mg (13-24 mg)
o 30 mg (25+ mg)
o Don't know

o Never taken



Turn Over

**For these herbals and other compounds, include pills, powders, tinctures, and teas taken regularly. Regularly means at least once a week for a year.** *(Mark all that apply.)*

**Co-enzyme Q10 (CoQ10)**

[illegible]
b) Days per week [illegible]
o Never taken

**DHEA**

o Take now / o Took only in past — a) Years taken in past 10 years [illegible]
b) Days per week [illegible]
o Never taken

**Fish oil, EPA, omega-3 or cod liver oil**

[illegible]
b) Days per week [illegible]
o Never taken

**Garlic pills**

o Take now / o Took only in past — a) Years taken in past 10 years: [illegible] o 3-5 o 6-10
b) Days per week: o 1-3 o 4-6 o 7
o Never taken

**Ginseng**

o Take now / o Took only in past — a) Years taken in past 10 years: o 1-2 o 3-5 o 6-10
b) Days per week: o 1-3 o 4-6 o 7
o Never taken

**Grapeseed, pycnogenol or proanthocyanidin**

o Take now / o Took only in past — a) Years taken in past 10 years [illegible]
b) Days per week [illegible]
o Never taken

**Melatonin**

o Take now / o Took only in past — a) Years taken in past 10 years [illegible]
b) Days per week [illegible]
o Never taken

**SAM-e**

o Take now / o Took only in past — a) Years taken in past 10 years [illegible]
b) Days per week [illegible]
o Never taken

**Saw palmetto**

o Take now / o Took only in past — a) Years taken in past 10 years: o 1-2 o 3-5 o 6-10
b) Days per week: [illegible] o 4-6 o 7
o Never taken

**Soy supplements (not milk) or isoflavones**

o Take now / o Took only in past — a) Years taken in past 10 years: o 1-2 o 3-5 o 6-10
b) Days per week: o 1-3 o 4-6 o 7
o Never taken

## LIFETIME SUPPLEMENT USE

9. **Have you *ever* taken any of these supplements at least once a week for a year?** *Mark all that apply.*

| | At least once a week for a year? | | Total years taken since age 21? 1-4 | 5-9 | 10-14 | 15-24 | 25+ |
|---|---|---|---|---|---|---|---|
| Multivitamins | o Yes | o No | o | o | o | o | o |
| Vitamin C (not in multivitamin) | o Yes | o No | o | o | o | o | o |
| Vitamin E (not in multivitamin) | o Yes | o No | o | o | o | o | o |
| Calcium (not in multivitamin) | o Yes | o No | o | o | o | o | o |



**Finished**

## Food Questionnaire and Food Preparation Questionnaire Instructions

If you have been diagnosed with prostate cancer, please answer the dietary questions to reflect your diet in the **12 months prior to your diagnosis**. **If you have not been diagnosed with prostate cancer**, please answer the diet assessment questions for the **12 months prior to this date**.

Please **answer all** questions within the questionnaire. Use the Food Questionnaire Serving Size Pictures page to aid in choosing the correct serving size. When answering the questions, please use a **#2 pencil** and completely fill-in the oval. An example is given below:

| | NEVER or less than once per month | 1 per month | 2-3 per month | 1 per week | 2 per week | 3-4 per week | 5-6 per week | 1 per day | 2+ per day | Medium serving size | S | M | L |
|---|---|---|---|---|---|---|---|---|---|---|---|---|---|
| Cold cereals | O | O | O | ● | O | O | O | O | O | 1 ½ cups | O | ● | O |
| Cooked cereals and grits | O | ● | O | O | O | O | O | O | O | 1 ½ cups | ● | O | O |
| Milk on cereals | O | O | O | ● | O | O | O | O | O | ¾ cups | O | ● | O |
| Pancakes, French toast and waffles | O | O | O | O | O | ● | O | O | O | 2 pieces | O | O | ● |

THIS IS AN EXAMPLE

# ***DO NOT USE INK***

## Supplement Use Questionnaire Instruction

Please **answer all** questions within the questionnaire. When answering the questions, please use a **#2 pencil** and completely fill-in the oval. An example is given below:

**Zinc in Multivitamin**

- ● Yes →
  - ● 15 mg (1-19 mg)
  - o 30 mg (20-49 mg)
  - o 60 mg (50-74 mg)
  - o 100 mg (75+ mg)
- o No

**Selenium in Multivitamin**

- o Yes →
  - o 25 mcg (1-39 mcg)
  - o 50 mcg (40-74 mcg)
  - o 100 mcg (75+ mcg)
- ● No

**Does your MULTIVITAMIN contain any of these other vitamins and minerals?**
*Mark all that apply.*

- ● Thiamin (B1)
- o Riboflavin (B2)
- o Niacin (B3)
- o Vitamin D
- o Magnesium
- o Copper
- o Chromium
- ● Iron

*****Please answer all questions that apply to you. Be sure to mark the "No" or "Never taken" responses if you do not use the listed vitamin, mineral, or supplement.*****

## Note: Questions are on both sides of the form

# Food Questionnaire Serving Size Pictures

**CaP Genes- Prostate Cancer Genetic Epidemiology Study**

Instructions For FFQ

Please use these pictures to help estimate your usual serving sizes.

- Medium serving sizes are given on the Food Questionnaire.
- A small serving is about one-half (1/2) the medium serving size or less.
- A large serving is about one-and-a-half (1 1/2) times the medium serving size or more.

**Beef, pork, chicken and fish as a main dish**

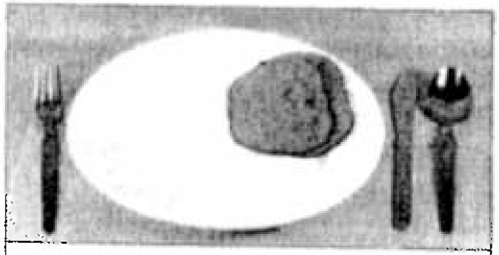
Small (3 ounces)

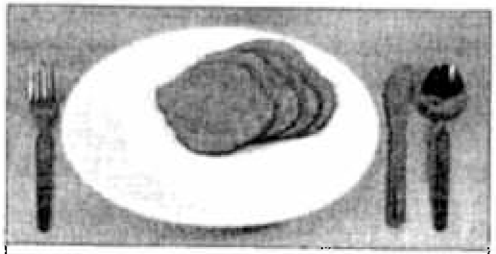
Medium (6 ounces)

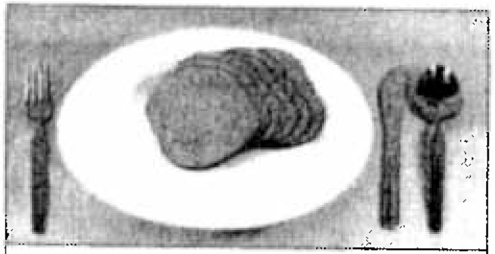
Large (9 ounces)

**Spaghetti and casseroles**

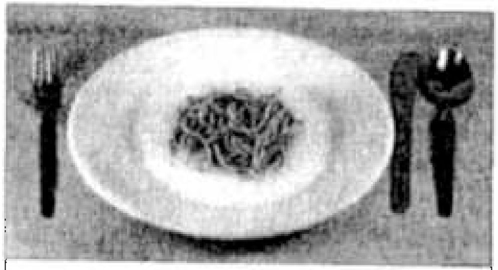
Small (3/4 cup)

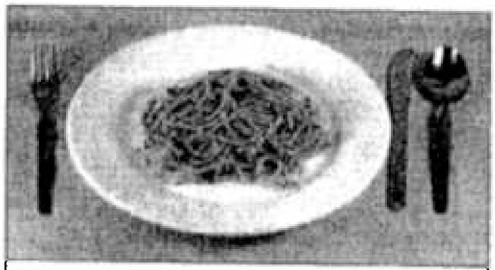
Medium (1 1/2 cups)

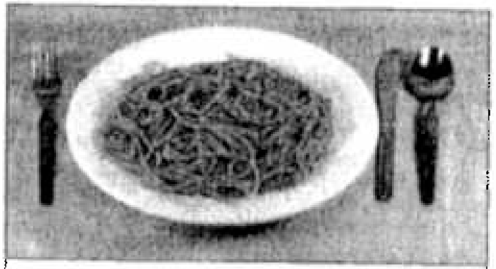
Large (2 1/2 cups)

**Vegetables such as green beans, corn, and potatoes**

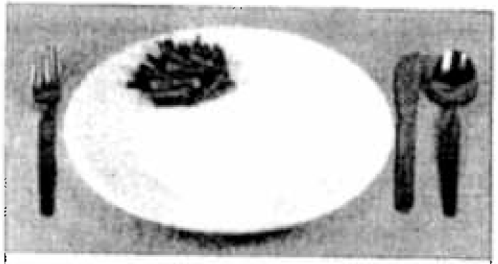
Small (1/3 cup)

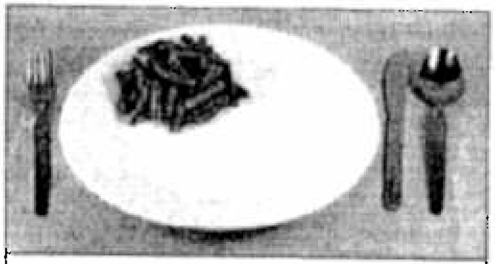
Medium (3/4 cup)

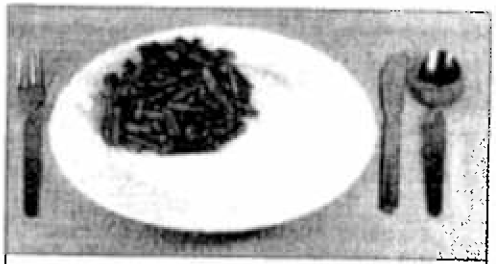
Large (1 1/2 cups)

# Food Preparation Questionnaire

The following questions are about foods that you ate **12 months prior to your prostate cancer diagnosis**. **If you <u>have not</u> been diagnosed with prostate cancer,** please answer the following questions for the **12 months prior to this date**.

1. **When you ate in restaurants during the previous year, did you eat grilled or barbecued meats?** (Grilled or barbecued meats are cooked *over* charcoal or a hot gas flame. Some fast-food restaurants call this "flame grilled". This does not include broiled or fried meats.)

   - o Never or less than once per month ⟶ **Skip to question 2**
   - o Yes

*****During the previous year, please report the average number of times you ate the following items. On average, indicate how well you cooked your meat for each of the applicable products below. If the meats were mainly from a fast food restaurant, then mark "Very well done".*****

| | Never or less than once per month | 1 per month | 2-3 per month | 1 per week | 2 per week | 3-4 per week | 5-6 per week | 1 per day | 2+ per day | <u>Usual</u> meat doneness | | | |
|---|---|---|---|---|---|---|---|---|---|---|---|---|---|
| | | | | | | | | | | Rare | Medium | Well done | Very well done |
| Beef (ribs, steaks, etc.) | o | o | o | o | o | o | o | o | o | o | o | o | o |
| Hamburgers (beef, turkey, vegetable, etc.) | o | o | o | o | o | o | o | o | o | o | o | o | o |
| Dark Fish (salmon, tuna, etc.) | o | o | o | o | o | o | o | o | o | o | o | o | o |
| Other Fish | o | o | o | o | o | o | o | o | o | | | | |
| Seafood | o | o | o | o | o | o | o | o | o | | | | |
| Chicken (with skin) | o | o | o | o | o | o | o | o | o | | | | |
| Chicken (without skin) | o | o | o | o | o | o | o | o | o | | | | |
| Pork (ribs, chops, etc.) | o | o | o | o | o | o | o | o | o | | | | |



**Turn Over**

2. **In the summer months during the previous year, did you eat meats cooked on an outdoor grill or barbecue?**

- o Never or less than once per month ⟶ **Skip to question 3**
- o Yes

*****On average over the previous year, please report the number of times you ate the following items and the way they were prepared*****

| | **Never or less than once per month** | 1 per month | 2-3 per month | 1 per week | 2 per week | 3-4 per week | 5-6 per week | 1 per day | 2+ per day | **Usual meat doneness** | | | |
|---|---|---|---|---|---|---|---|---|---|---|---|---|---|
| | | | | | | | | | | **Rare** | **Medium** | **Well done** | **Very well done** |
| Beef (ribs, steaks, etc.) | o | o | o | o | o | o | o | o | o | o | o | o | o |
| Hamburgers (beef, turkey, vegetable, etc.) | o | o | o | o | o | o | o | o | o | o | o | o | o |
| Hot dogs (beef, turkey, etc.) | o | o | o | o | o | o | o | o | o | N/A | N/A | o | o |
| Dark Fish (salmon, tuna, etc.) | o | o | o | o | o | o | o | o | o | o | o | o | o |
| Other Fish | o | o | o | o | o | o | o | o | o | | | | |
| Seafood | o | o | o | o | o | o | o | o | o | | | | |
| Chicken (with skin) | o | o | o | o | o | o | o | o | o | | | | |
| Chicken (without skin) | o | o | o | o | o | o | o | o | o | | | | |
| Pork (ribs, chops, etc.) | o | o | o | o | o | o | o | o | o | | | | |

3. **During the previous year did you eat smoked meats?**

- o Never or less than once per month ⟶ **Finished**
- o Yes

| | **Never or less than once per month** | 1 per month | 2-3 per month | 1 per week | 2 per week | 3-4 per week | 5-6 per week | 1 per day | 2+ per day |
|---|---|---|---|---|---|---|---|---|---|
| Smoked ham | o | o | o | o | o | o | o | o | o |
| Smoked turkey | o | o | o | o | o | o | o | o | o |
| Smoked fish | o | o | o | o | o | o | o | o | o |
| Other smoked meats | o | o | o | o | o | o | o | o | o |



**Finished**

# Chronological Dietary Questions Instructions

Please use a **pen or a pencil** to mark the appropriate responses. Below is an example:

1) Is your present diet different from your diet when you were in your 40's?

_√_ Yes
___ No (Go to the next section)

2) Was your meat intake in your 40's…

___ Much greater than now
_√_ Slightly greater than now
___ The same as now
___ Slightly less than now
___ Much less than now

Refer to the dotted line with diamond ends (◆------◆) when answering the questions on page 1.
Refer to the solid line with arrow ends (◀——▶) when answering the questions on page 2.

**Note: Questions are on both sides of the form**

# Chronological Dietary Questions Timeline

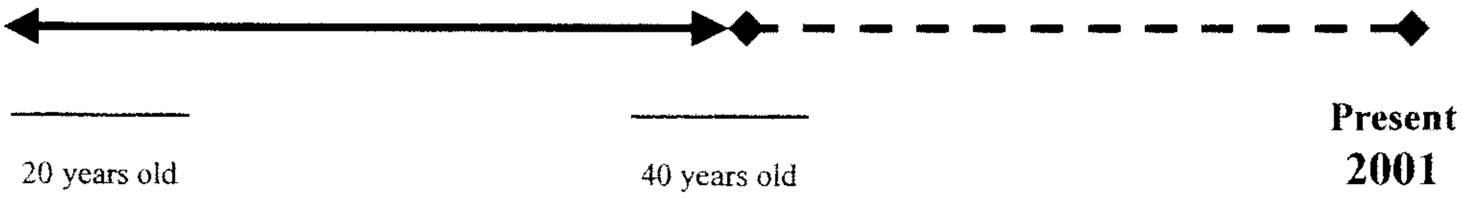


**Please fill-in the years when you were 40 and 20 years old**

# Chronological Dietary Questions

1) Is your present diet different from your diet when you were in your 40's?

___ Yes
___ No (Please go to Question 7)

2) Was your meat intake in your 40's...

___ Much greater than now
___ Slightly greater than now
___ The same as now
___ Slightly less than now
___ Much less than now

3) Was your vegetable intake in your 40's...

___ Much greater than now
___ Slightly greater than now
___ The same as now
___ Slightly less than now
___ Much less than now

4) Was your fruit intake in your 40's...

___ Much greater than now
___ Slightly greater than now
___ The same as now
___ Slightly less than now
___ Much less than now

5) Was your sodium intake in your 40's...

___ Much greater than now
___ Slightly greater than now
___ The same as now
___ Slightly less than now
___ Much less than now

6) Was your cholesterol intake in your 40's...

___ Much greater than now
___ Slightly greater than now
___ The same as now
___ Slightly less than now
___ Much less than now



**Please Turn Over**

# Chronological Dietary Questions

7) Was your diet when you were in your 40's different from your diet when you were in your 20's?

___ Yes
___ No (This section is completed)

8) Was your meat intake in your 20's...

___ Much greater than your 40's
___ Slightly greater than your 40's
___ The same as your 40's
___ Slightly less than your 40's
___ Much less than your 40's

9) Was your vegetable intake in your 20's...

___ Much greater than your 40's
___ Slightly greater than your 40's
___ The same as your 40's
___ Slightly less than your 40's
___ Much less than your 40's

10) Was your fruit intake in your 20's...

___ Much greater than your 40's
___ Slightly greater than your 40's
___ The same as your 40's
___ Slightly less than your 40's
___ Much less than your 40's

11) Was your sodium intake in your 20's...

___ Much greater than your 40's
___ Slightly greater than your 40's
___ The same as your 40's
___ Slightly less than your 40's
___ Much less than your 40's

12) Was your cholesterol intake in your 20's...

___ Much greater than your 40's
___ Slightly greater than your 40's
___ The same as your 40's
___ Slightly less than your 40's
___ Much less than your 40's



**Finished**
